# Fostering Sustainability through Visualization Techniques for Real-Time IoT Data: A Case Study Based on Gas Turbines for Electricity Production

**DOI:** 10.3390/s20164556

**Published:** 2020-08-14

**Authors:** Ana Lavalle, Miguel A. Teruel, Alejandro Maté, Juan Trujillo

**Affiliations:** 1 Lucentia Research, DLSI, University of Alicante, Carretera San Vicente del Raspeig s/n, 03690 Alicante, Spain; materuel@dlsi.ua.es (M.A.T.); amate@dlsi.ua.es (A.M.); jtrujillo@dlsi.ua.es (J.T.); 2 Lucentia Lab, Avda. Pintor Pérez Gil, N-16, 03540 Alicante, Spain

**Keywords:** Internet of Things, data visualization, Big Data analytics, sustainable production, gas turbines, Artificial Intelligence

## Abstract

Improving sustainability is a key concern for industrial development. Industry has recently been benefiting from the rise of IoT technologies, leading to improvements in the monitoring and breakdown prevention of industrial equipment. In order to properly achieve this monitoring and prevention, visualization techniques are of paramount importance. However, the visualization of real-time IoT sensor data has always been challenging, especially when such data are originated by sensors of different natures. In order to tackle this issue, we propose a methodology that aims to help users to visually locate and understand the failures that could arise in a production process.This methodology collects, in a guided manner, user goals and the requirements of the production process, analyzes the incoming data from IoT sensors and automatically derives the most suitable visualization type for each context. This approach will help users to identify if the production process is running as well as expected; thus, it will enable them to make the most sustainable decision in each situation. Finally, in order to assess the suitability of our proposal, a case study based on gas turbines for electricity generation is presented.

## 1. Introduction

Global energy consumption is increasing on a daily basis [1,2]. New lifestyle trends are increasing the need for electricity generation. In order to cope with this ever-growing need, a sustainable energy production process is required [3]. In this sense, one approach to aiding the sustainability of energy production is to exploit the potential of the Internet of Things (IoT). The adoption of IoT by industry has led to highly sensorized machinery [4]. Thus, thanks to the data provided by these sensors, it is possible to better understand how an electricity production process is performing, and thus it is possible to take actions aimed at improving the throughput and sustainability of the whole process [5].

The introduction of Artificial Intelligence (AI) processing data provided by sensors has enabled the determination of whether a generation process is running as well as expected [6]. Indeed, Predictive Machine Learning can be applied in order to assess whether or not machinery may fail in the near future [7]. Nevertheless, such techniques are often based on the usage of neural networks, whose input is usually the general status (or a subset) of the whole system (i.e., tuples of the data generated from all the system’s sensors) [8]. Thus, since neural networks act as a black box, it is unlikely that they can provide information regarding the part of the system which is going to cause the predicted failure [9].

However, even if the output of the neural network can only determine whether the process is going to fail or not, the information of the production process can be complemented with visual details regarding the evolution of the machinery sensors. Thanks to these visualizations, machinery operators can identify abnormalities in certain parts of the system, enabling them to identify certain problems which could not be detected otherwise. Still, the creation of these visualizations is not trivial; the large volume of sensor data produced across multiple magnitudes makes it challenging to present the necessary information to users without making it overbearing.

Therefore, in order to make such visualizations possible, we propose a new methodological approach to monitor industrial machinery using an IoT-based visualization technique. The main goal of this work is to help non-expert users in data visualization to visually locate and understand the failures that could arise in a production process, thus enabling them to make the most sustainable decision in each situation.

In previous works [10,11], we defined a model that helped users to specify and achieve their goals. It extracted the characteristics of the data sources and automatically derived the best type of visualizations according to the defined context. Moreover, in [12], we have published an approach that is focused on the context of Smart Cities; in this work, we proposed a methodology, based on visualization techniques, with the aim of improving the evidence-gathering process by assisting users in their decision making in the context of Smart Cities.

In this paper, we have significantly improved and complemented our previous works as follows: (i) we show how our proposal can be applied to the context of industrial machinery, (ii) we broaden our proposed metamodel by adding new elements to make it suitable for real-time scenarios, and (iii) we provide a novel methodology to monitor industrial machinery, which is divided into two phases (the first phase is performed before runtime, and the second phase is executed at runtime). This methodology (a) to define the goals and requirements of the production process, (b) automatically derives the most suitable visualization type for each context, (c) helps users to visually understand the output of Artificial Intelligence models and (d) provides visualizations to help users to make the most sustainable decision in each situation.

**Figure 1:**
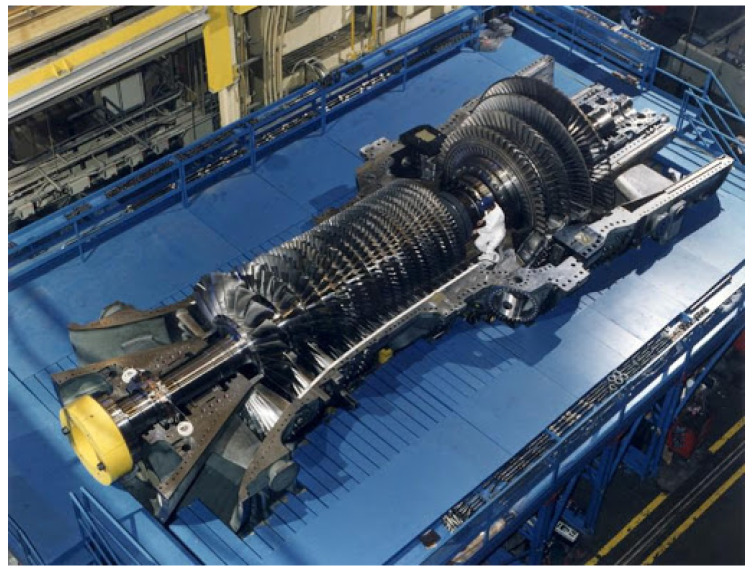
Gas turbine.

Furthermore, in order to test and show the applicability of our proposal, we have presented a case study based on gas-based electricity generation turbines. Gas turbines are large machines that can be heavily sensorized. A picture of a gas turbine can be seen in Figure 1. The gas turbine in our case study includes 80 sensors from which data are gathered at runtime. The complexity of the data, the speed at which data are generated and the importance of detecting failures make this a perfect scenario to test how our approach improves the sustainability of the process; i.e., how it improves the performance of the process by preventing the breakdown of the machines. The results of this case study confirm that our proposal helps to improve the sustainability of the process.

The advantages of our proposal are that (i) it enables users to monitor the quality of the systems, (ii) it aids in preventing the breakdown of the machines, (iii) it helps to identify if the production process is running as well as it was expected, and (iv) it helps users to understand and co-relate the outputs of an AI engine. Without the benefits introduced by our proposal, users would find it more difficult to determine the optimality of the execution of the production process. Moreover, it would be difficult for them to identify whether stopping production is the most sustainable decision.

The rest of the paper is structured as follows. Section 2 presents the related work in this area. Section 3 describes our proposed methodology for fostering sustainability through visualizations. Section 4 shows our approach, which is applied in a real case study for electricity generation based on gas turbines. Section 5 summarizes the limitations of our work. Finally, Section 6 summarizes the conclusions and sketches our future work.

## 2. Related Work

The importance of the sustainable development in industry is increasing. In 1992, the concept of sustainable production emerged at the United Nations Conference on Environment and Development [13]. There, it was determined that the main cause of the deterioration of the global environment is the unsustainable pattern of consumption and production, especially in industrialized countries.

The sustainability strategy includes indicators giving a measurable overview of trends and involves action by all sectors, especially industrial systems. This sector should play an important part in the attainment of sustainability goals [14]. The set of strategic metrics for assessing sustainability includes [15] (i) reflecting the status of a system, (ii) providing early warning information, (iii) anticipating future conditions and trends, (iv) comparing across places and situations and (v) highlighting what is happening in a large system.

In [16], a new methodology was presented to promote and measure sustainable production in business. The authors proposed 22 indicators and provided guidance to select additional, production-specific indicators.

As [17] argues, visualizations may help in making energy-saving management decisions. A visualization of the incoming data can provide insights. However, visualizing big data in real-time is a challenge itself. The growth of the Internet of Things (IoT) means that the amount of available real-time data is increasing rapidly; therefore, the development of analysis programs for IoT platforms is a complex task [18].

Cyber-physical systems are successful in various scientific communities, specifically regarding production issues [19]. The industry represents a rich data environment, and increasingly large volumes of data are constantly being generated by its processes. However, only a relatively small portion of the data is actually exploited by manufacturers [20].

Several works have focused on IoT visualization. For example, in [17], a platform is proposed to transform sensor data to context-based visualized data. One sector in which the visualization of IoT sensors is used is in the Smart Cities domain. These systems generate massive amounts of data that can be analyzed and visualized to better understand people’s dynamics [21]. Another sector is healthcare: the visualization of data, metadata and sensor networks is becoming one of the most important aspects of the health monitoring process [22]. In [23], an intelligent healthcare framework based on IoT technology is proposed, providing ubiquitous healthcare to users during their workout sessions. In [24], the authors propose an ambient intelligence environment for cognitive rehabilitation at home, combining physical and cognitive activities. They implement a Fuzzy Inference System in which smart sensors and actuators attempt to compensate for the absence of the therapist.

The visualization of a large data set is a demanding task. The traditional manners of presenting data face a few limitations as the amount of data grows constantly. In [25], the authors identified challenges in big data visualizations, such as perceptual scalability, real-time scalability and interactive scalability. They argue that visualization tools and techniques are able to help users in the identification of missing, erroneous or duplicate values.

The authors in [26] contribute methods for the visualization of big data in real-time. They present techniques to address perceptual and interactive scalability, following the principle that scalability should be limited by the chosen resolution of the visualized data, rather than the number of records. In [20], an Intelligent Data Analysis and Real-Time Supervision (IDARTS) framework is proposed that combines distributed data acquisition, machine learning and run-time reasoning to assist in fields such as predictive maintenance and quality control. The goal of their framework is to allow manufacturers to translate their data into a business advantage.

In [18], the authors present I${}^{2}$—an interactive development environment that coordinates running cluster applications and corresponding visualizations, where only the currently depicted data points are processed and transferred. They present a model for the real-time visualization of time series and show how cluster programs can adapt to changed visualization properties at runtime to enable interactive data exploration on data streams. Additionally, [27] presented Hashedcubes—a data structure for answering queries from interactive visualization tools that explores and analyzes large, multidimensional datasets. This enables the real-time visual exploration of large datasets with low memory requirements and low query latencies.

The aforementioned works highlight the importance of the use of visualizations in IoT scenarios. On the other hand, other works such as [28] highlight the importance of fault detection and isolation in safety-critical systems, such as gas turbine engines. They discuss the necessity of a decision-support system to prescribe corrective actions so that the system can continue to function without jeopardizing the safety of the personnel and equipment involved. The authors [28] propose the use of Self-Organizing Maps (SOM) in order to visually explore the data in a two-dimensional space, understand the nature of the input signal and gain insights into the difficulty of the fault classification task. SOM transforms complex, nonlinear relationships between high-dimensional data into topological relationships in a low-dimensional space.

Other works, such as [29], visualize turbulent flow behavior between turbines in a physical space and allow the viewer to see intricate vortex-blade intersection configurations in a static-blade view. In [30], examples of the implementation of optical techniques employed to visualize flow structure, fuel spray patternation, liquid fuel penetration and combustion species are presented.

In [31], an OSRDP architecture framework for sustainable manufacturing is proposed. The authors propose a system that is capable of processing massive sensor data efficiently when the amount of sensors, data and devices increases. The system uses data mining based on Random Forest to predict the quality of products. However, the proposed system classifies sensors as normal/abnormal on an individual basis; it does not take into account problems that are only reflected by the readings of the system as a whole. Moreover, it does not analyze which visualizations would be most adequate to troubleshoot the underlying problems, making it more difficult to make adequate decisions for their correction.

One of the core benefits of visualizations is that it enables people to discover visual patterns that might otherwise be hidden [32]. However, it is very important to be mindful of which types of visualizations are used in each context. Not all types of visualizations are suitable for visually detecting anomalies; as [32] discusses, it is possible to create visualizations that seem “plausible” (design parameters are within normal bounds and pass the visual sanity check) but hide crucial data features.

As we have shown, different approaches highlight the importance and challenges of visualizing real-time data from IoT systems. Other approaches highlight the importance of systems that detect and predict failures in order to achieve sustainable production. However, none of the approaches listed above provide a complete methodology that captures information from an IoT system in order to predict when the system may potentially fail and enables users to make the most sustainable decision with the aid of real-time visualizations.

Therefore, we propose a methodology that chooses the best type of visualization based on users’ analytical needs. Moreover, visual techniques are provided so that users can understand the output of Artificial Intelligence models. This will enable users to monitor the quality of the systems and to make the most sustainable decision in each situation.

## 3. Methodology to Foster Sustainability through Visualizations

Once the related work has been presented, this section will describe our methodology. The main aim of our proposal is to help users to visually locate and understand the failures that could arise in a production process. Our methodology includes two phases. Phase 1 is the setup phase, performed before production (runtime). In this phase, users define the goals and requirements of the production process; this information is used to generate the best suited visualizations. Phase 2 is executed during the production process (at runtime). In this phase, the production process is monitored with the objective of aiding users in making the most sustainable decisions. In the following, we describe these two proposed phases in detail.

### 3.1. Phase 1—Definition of Goals and Visualizations

As mentioned above, Phase 1 is executed prior to the production process. The objective of this phase is for users to define the goals that they are aiming to achieve during the production process. Therefore, the most proper type of visualization to achieve these goals will be automatically derived. These visualizations, defined in the pre-production process, will be used to detect and monitor failures in the production process. In this sense, we ensure that the visualizations shown to users are the most suitable to meet their goals and help them to make decisions about the production process.

**Figure 2:**
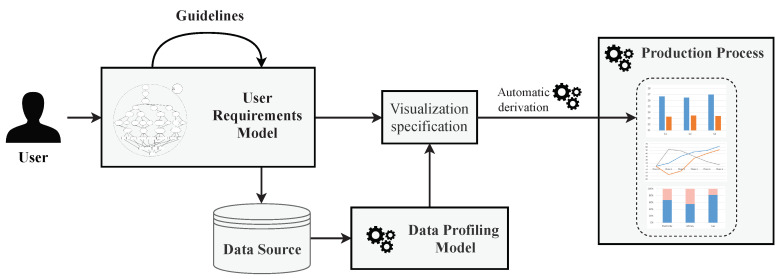
Phase 1—visualization definition process.

Figure 2 summarizes the process followed in Phase 1, which defines visualizations. Firstly, users create a User Requirements Model aided by a sequence of guidelines published in [10]. This model guides non-expert users to capture their analytical needs. Furthermore, through this User Requirements Model, users define, among others, which elements of the data source they wish to represent in the visualizations. Complementary to this model, a Data Profiling Model [10] is obtained by analyzing the features of the data sources to be visualized in a semi-automatic manner.

Once both models have been obtained, they are translated into a visualization specification. Following [33], we are able to derive the visualization specification into the most suitable visualization to achieve each specified goal in an automated manner.

These generated visualizations are introduced in the production process at each defined moment. Therefore, users will be able to monitor the production and make decisions more accurately based on the visualizations. In the following, we describe the elements included in the visualization definition orocess.

#### 3.1.1. User Requirements Model

Our approach starts from a User Requirements Model that guides non-expert users towards the definition of specific visualizations that they would need to achieve their data analysis objectives. It is possible to find an example of the User Requirements Model applied to a real case in Section 4.

**Figure 3:**
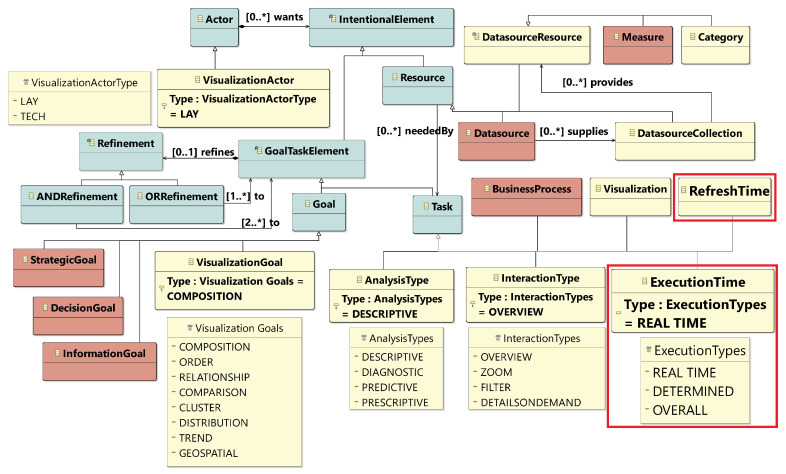
User Requirements Metamodel.

In order to formally define our novel model, we propose a metamodel (see Figure 3). This metamodel is an extension of the model used for social and business intelligence modeling [34], namely i* [35] and the i* for data warehouses extension [36]. It is worth noting that i* has already been extended and used to model other real-time IoT-enabled domains [37].

In Figure 3, elements from i* are represented in blue, elements from i* for data warehouses are represented in red and the elements added in our proposal are in yellow, including the new elements introduced to work with real-time scenarios (represented within a red square). In the following, we will describe the elements of the metamodel.

The user of the system is represented with the visualization actor element. We can find two types of visualization actors: lay, when the user is not expert in complex data visualizations, or tech, when the user has experience in data visualization. The next element is the business process on which users will focus their analysis. This process will serve as a guideline for the definition of different goals.

Then, the analysis type enables users to define which kind of analysis they want to perform. In order to determine the type of analysis, the user may select which of the following questions [38] needs to be answered: (prescriptive) How to act? (diagnostic) Why has it happened? (predictive) What is going to happen? or (descriptive) What should be done to make it happen?

The visualization element represents a visualization type that will be created to satisfy the visualization goals. The aspect of the data that the visualization should describe is represented with the visualization goal. These goals can be defined as comparison, trend, relationship, composition, cluster, geospatial, distribution, order or cluster, as considered in [33]. Furthermore, the visualizations have one or more interaction type; this element represents the interaction that the user aims to have with the visualization. As considered in [33], the different kinds of interaction are the following: details on demand, zoom, overview or filter. Finally, a visualization will make use of a datasource resource, which will feed the data to the visualization.

Furthermore, in order to cope with real-time scenarios, we have added new elements that capture the execution time and the refresh time. The execution time element defines whether the visualization will be executed in real-time, at a specific moment, or if it shows an image of the overall process, while the refresh time element defines the interval of time in which the visualization will be updated.

**Figure 4:**
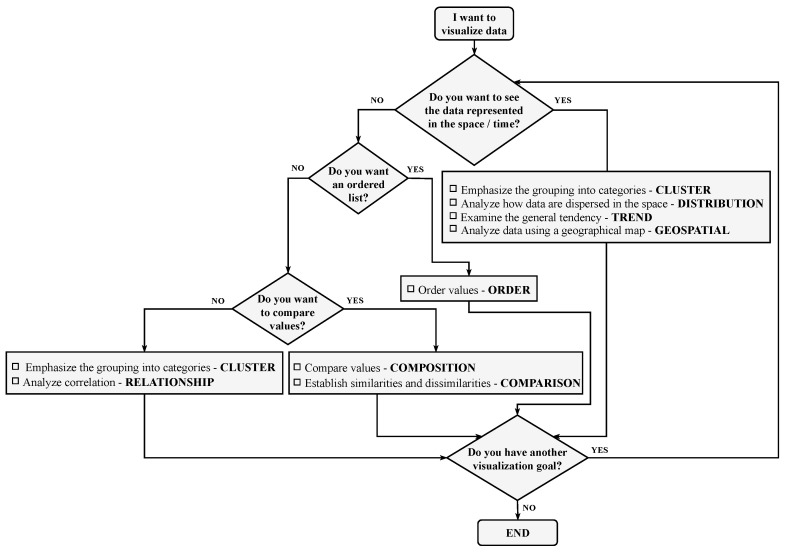
Guidelines expressed as a flowchart to help non-expert users to define visualization goals.

As argued in [33], it can be difficult for non-expert users to give proper values to these elements. For example, choosing the correct visualization goal can be difficult. Therefore, our proposal includes some guidelines as shown in the flowchart in Figure 4. This element helps users to choose which visualization goal best suits their needs. In [10], we propose other alternatives to make the definition of model elements easier for non-expert users.

#### 3.1.2. Data Profiling Model

The next model involved in the process is the Data Profiling Model; this model captures characteristics of the data that are relevant for visualization. Firstly, through the User Requirements Model, users select the data elements that they want to represent in the visualizations. Then, through the Data Profiling Model, the data characteristics of dimensionality, cardinality and dependent/independent type are extracted in a semi-automatic manner, as explained below.

- Cardinality can be defined as low or high, depending on the number of items it is necessary to represent. Low cardinality is defined as when there are few dozens of items to represent, while high cardinality is when there are several dozens of items or more.
- Dimensionality represents the number of variables to be visualized. It can be defined as one-dimensional when the data to represent are a single numerical value or string, two-dimensional when one dependent variable depends on one independent variable, n-dimensional if each data object is a point in an n-dimensional space, Tree if a collection of items have a link to one parent item, or graph when a collection of items is provided and each item is linked to an arbitrary number of other items.
- The type of data defines the data type of each variable. It can be defined as nominal if each variable is assigned to one category, ordinal when each variable is assigned to one category and the categories can be sorted, interval when it is possible to determine the equality of intervals or ratio when there is a unique and non-arbitrary zero point.

#### 3.1.3. Derivation of Visualizations

Once the User Requirements Model and the Data Profiling Model are completed and all the requirements have been gathered, a visualization specification can be built. This process is covered in [11], where the transformation from a visualization specification into a visualization implementation is performed following a Model-Driven Architecture (MDA) standard.

### 3.2. Phase 2—Monitoring of Production Process

**Figure 5:**
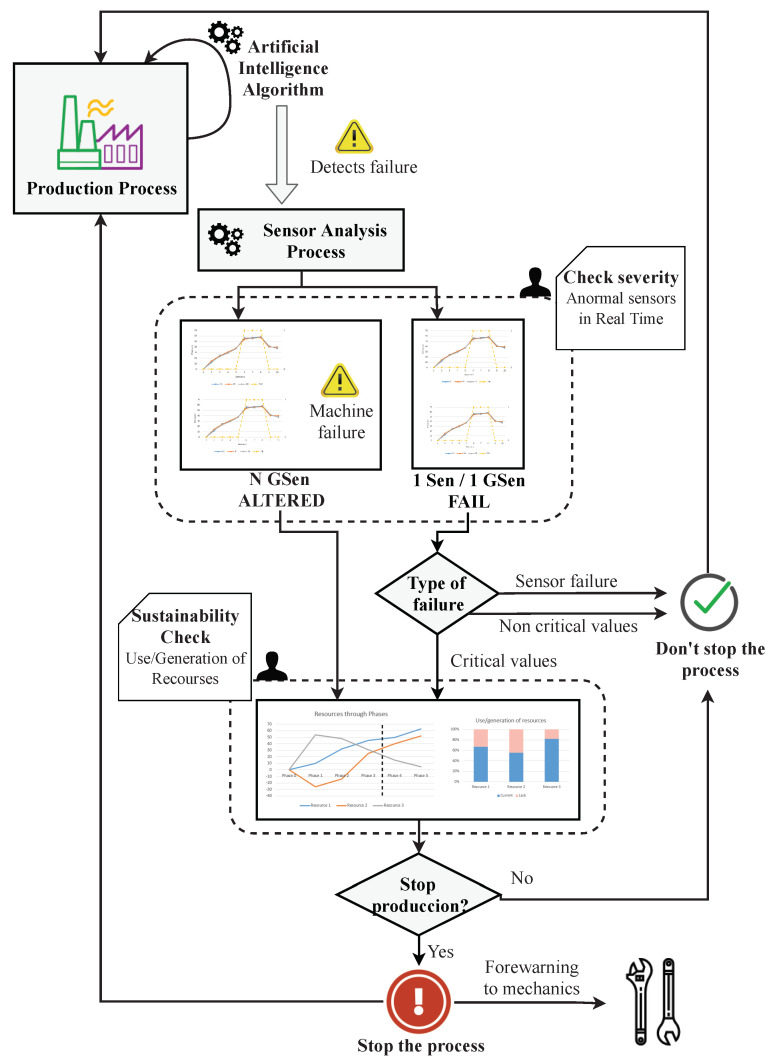
Phase 2—production process.

Once Phase 1 is completed, users will have defined their goals. Furthermore, the best types of visualization to achieve and measure their goals will have been proposed. Then, the production process starts. Figure 5 summarizes the approach to the production process in our proposal. In the figure, we can see how visualizations generated through the visualization definition process (Figure 2) are integrated and how users intervene during the severity and sustainability check in order to decide whether the production should be stopped or not. In the following, we describe the different components depicted in Figure 5 in more detail.

#### 3.2.1. Cloud Computing Architecture

**Figure 6:**
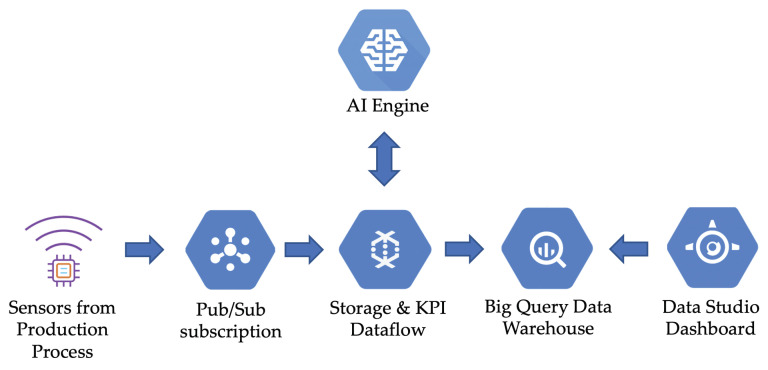
Cloud computing architecture of the system.

In order to integrate the real-time data from the sensors with the final dashboard, we have designed the Cloud computing architecture shown in Figure 6. Firstly, the data from the sensors in the production process are collected through a Pub/Sub queue. After that, a streaming analysis pipeline will read the data from the queue and send the data to the AI Engine. Then, the data from the sensors, along with the output data from the Artificial Intelligence model, are stored in a data warehouse. From this data warehouse, the visualizations are fed by the data to be represented in the dashboards that will be presented to the final user.

#### 3.2.2. Artificial Intelligence Model

The first element of the process (Figure 5) is an Artificial Intelligence model. This element is used to detect if there is any potential failure in the production process. A detailed explanation regarding how predictive neural networks work is beyond the scope of this paper. Our proposal is focused on providing techniques to visually understand the output of the models; however, we will briefly explain how these models work together in order to make our proposal more comprehensible.

**Figure 7:**
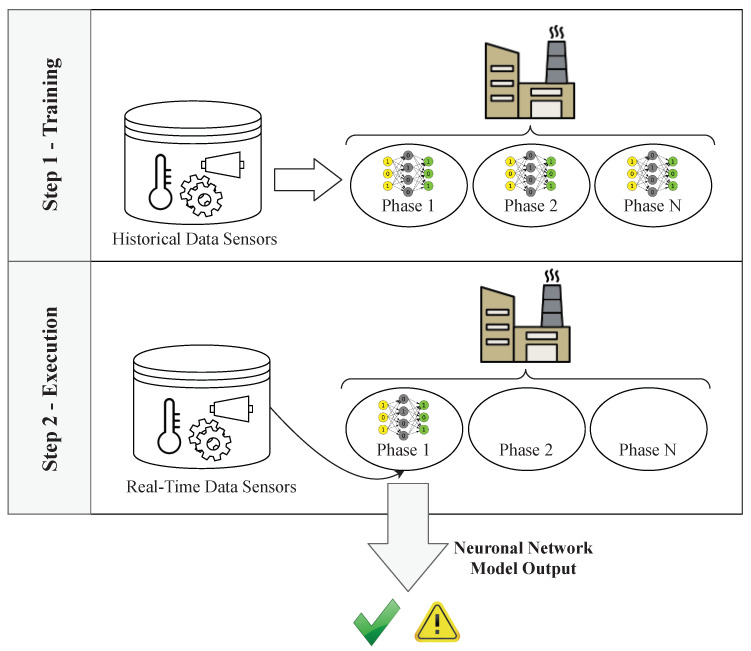
Artificial Intelligence model.

As Figure 7 shows, the first Artificial Intelligence model is fed with data from the different sensors of the process and divided into two steps. Firstly, as Step 1 in Figure 7 shows, a clustering algorithm is used [39]. This kind of algorithm analyzes the incoming data from the sensors in order to differentiate the phases that compose the production process by analyzing the different values that the sensors have in the whole process. Therefore, the output of this algorithm will be a model for the definition of the phases that compose the process.

Once the phases have been identified, a Deep Neural Network [40] based on Variational Autoencoders (VAEs) for anomaly detection [41] is trained in each cluster (phase). Once the neural network is trained, as Step 2 in Figure 7 shows, the data from the values of the sensors are analyzed in real-time. First, the incoming data are analyzed by the clustering model in order to discover the phase in which the data have been generated. Once the phase is identified, the neural network corresponding to that phase is called for prediction. This neural network identifies whether there are potential failures present in the production process. Therefore, the output will be a data tuple encoded by the corresponding VAE. The Euclidean distance between input and output tuples will be used to assess whether or not the input of the model corresponded to an anomalous situation of the machinery.

With the information provided from the neural network and the clustering model, users are able to determine if a potential failure has been detected, as well as the phase of the process in which it was detected. However, due to the black-box nature of neural networks, this information is insufficient to understand the root cause of the problem. Therefore, our approach introduces the next element: the sensor analysis process.

#### 3.2.3. Sensor Analysis Process

Once the Artificial Intelligence model has detected that there is a potential failure in the process, the sensor analysis process (see Figure 5) enables users to detect what type of fault has occurred in real-time and make decisions according to the severity of the problem.

The sensor analysis process compares the values of the sensors in order to detect which are out of range. There are two situations in which our system detects a failure: on the one hand, our proposal defines that a sensor is out of range when the current value exceeds the limits defined in its hardware specification; on the other hand, from time to time, a system failure is not produced by the failure of an individual sensor—in these cases, the fault is identified by the anomalous values of a set of sensors. These sensors may have individual values within adequate operation ranges; however, their combined status can be abnormal with regards to the production process. As an example of this situation, an energy-generation engine’s throughput sensor could send a value of 1% while a related temperature sensor could be measuring 300 °C. Despite both measurements being correct according to their hardware specifications, it is illogical that an engine could work at that capacity while having such a high temperature. Thus, taking into account both of the explained scenarios, our approach covers them as follows.

- N GSen ALTERED (Machine failure): N groups of sensors are altered. An alteration means that there is a small alteration in the values of the sensors but that no sensor is out of its acceptable ranges. Therefore, groups of visualizations are generated. These visualizations represent all sensors of the machine, grouped by the unit of measure and the localization in the machine. Furthermore, warnings will be considered, thus warning users that the machine is presenting an abnormal status and that it is possible that the production optimal.
  When this scenario arises, additional information will be necessary in order to make decisions. This new information will help users to decide if, at that moment, it is sustainable to stop the production or not.
- 1 Sen/1 GSen FAIL: There is one sensor or a group of sensors which is out of range. In these cases, a group of visualizations are generated in which the anomalous sensor/sensors with their real-time values are represented, split by the unit of measurement. Furthermore, in order to display a reference, the historical average value of these sensors is also represented. Moreover, these visualizations include the values of sensors located physically close to the relevant sensor which do not present anomalies.
  When this case arises, users should make their first decision. As Figure 5 shows, users should decide, relying on the visualizations, if the failure is a device failure or is not critical. Otherwise, they must decide if it is a critical moment and therefore necessary to consider the possibility of stopping the production process.
  – Sensor failure or non-critical values: If users decide that the failure is caused due to a broken sensor or if the values that the sensor is showing are acceptable or are located in non-critical areas, the production process will continue. However, if users deem it necessary, it is possible to use the visualizations to continuously monitor the values of these abnormal sensors, thus allowing users to visualize the values of these sensors in real-time and take measures if at any time the sensors reach critical values.
  – Critical values: If users decide that the values of the sensors are critical fir the production process, it will be necessary to present additional information in order to help to users to decide if, at that moment, it would be sustainable to stop the production or not.

After this process, users are able to check the severity of failures during the production process and locate the problem by analyzing the sensors through visualizations. Furthermore, if users detect a severe problem, more information will be shown so that they will be able to decide whether it is sustainable to stop the production at that moment or not. In the following section, we describe this sustainability check in more detail.

#### 3.2.4. Sustainability Check

The sustainability check (see Figure 5) is performed when users have detected that there is a potential critical failure in the production process. Therefore, at that moment, users need more information in order to decide whether it would be sustainable to stop production or not. They can decide if it is more optimal that production continues with some risk of failure, even if sensors or some machinery pieces may be damaged. In order to make these decisions, a set of visualizations is needed that measure the used/generated resources at each phase of the production process, enabling users to analyze the situation and make decisions according to the expected consequences.

Thanks to the application of the AI models, we are aware of the exact phase which the production process has reached. With this knowledge, a set of visualizations are generated by following the design defined in phase 1 of the process (Figure 2), in which the visualizations required to achieve user goals were derived. This visualizations present the expected evolution of the system in terms of costs, risks and resources. For example, users may decide that during the initial phase, many resources have been spent and the production has been low. Therefore, it would not be sustainable to stop the production at this moment since the cost would be too high. However, during a more advanced phase, the resources spent have already been amortized, and stopping the production at this moment will lead to an acceptable reduction of the profit without significant resource losses.

Once users have analyzed the visualizations, if they decide to avoid stopping the production, the affected parts of the machinery will be monitored. The system will create a very detailed visualization of the sensors of each part of the machinery in order to enable users to stop the process at the moment at which the sensors reach critical values. This could potentially avoid risky situations for the machinery as well as for the corresponding operators.

In the case that users decide to stop the production process, a forewarning will be sent to the mechanics with all the information of the affected parts and the values of the sensors. Therefore, the mechanics will be able to study the cause of the failure and will be able to intervene as soon as production is completely stopped in order to make the necessary repairs.

## 4. Case Study: Gas Turbines for Electricity Generation

This scenario has been developed in the context of an international project under a non-disclosure agreement (NDA). Since the data are industrial property, we provide real data in an anonymized manner and thus do not provide details of the turbine or specific sensors. Moreover, the data shown in this work have been altered to avoid presenting real data protected by the NDA.

In the following, we show how our approach is applied to a real case study of a company that produces electricity using gas turbines. The main goal of the company is to improve the sustainability of the process. In order to achieve this goal, the company requires a set of visualizations to analyze their data in real-time in order to foster the decision-making process regarding when it is optimal and sustainable to stop the production process at a given point in time. The gas turbines for electricity generation used in this case of study consist of 80 sensors, from which data are gathered at runtime. These sensors are located along the machine and measure all relevant magnitudes, including the temperature, pressure, frequency, speed, humidity, etc. of different parts of the gas turbine. Some of them are replicated to ensure correct measurements.

Following the Cloud computing architecture shown in Figure 6, in this specific case study, we used Google Cloud Dataflow to collect and process the data from sensors in real-time. These data, as well as the information from the output of the Artificial Intelligence models, have been stored into a BigQuery data warehouse. Finally, we have chosen Google Data Studio to perform the visualizations.

### 4.1. Phase 1—Definition of Goals and Visualizations

**Figure 8:**
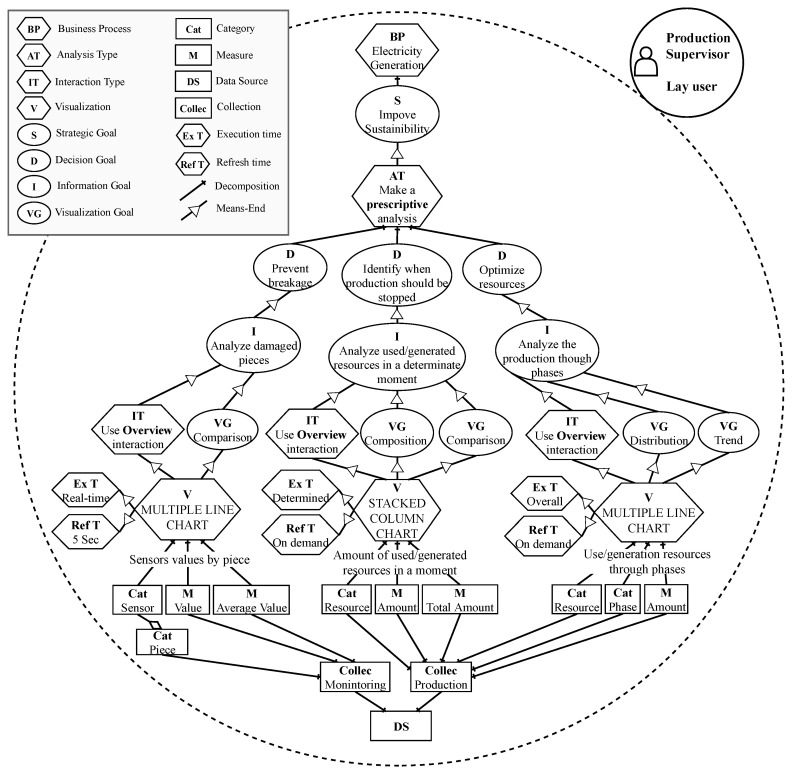
Application of our User Requirements Model to the case study.

Following the application of our approach (Figure 2), the first step in phase 1 is to create a User Requirements Model. In Figure 8, we can see the result of its application. In this case, the user is a production supervisor; however, this user is not an expert in data visualization. Therefore, the user is defined as a “lay user”, and the analysis will therefore be focused on the “Electricity Generation” business process.

Next, the strategic goal is defined as “improve sustainability”, and the type of analysis to perform is “prescriptive analysis”, meaning that the user wants to know how to act in the process; specifically, whether the process should be stopped or not.

The prescriptive analysis is decomposed into decision goals. These goals are defined by the user as “prevent breakage”, “identify when production should be stopped”, and “optimize resources”. By themselves, the decisions goals do not provide the necessary details about the data to be visualized. Therefore, for each decision goal, the user has to specify information goals.

From each of the decision goals, the user decided upon the following information goals: “analyze damaged pieces”, “analyzed used/generated resources at a certain moment” and “analyze the production though phases”. For each information goal, one visualization will be created to achieve it.

The user defines the visualization goals following the guidelines shown in Figure 4. In this case, the user defines “comparison”, “composition”, “distribution” and “trend” as visualization goals. The user also defines the kind of interaction that they would like to have in the visualization as “overview”. Furthermore, since we are using a real-time scenario, the user must define the execution and refresh time of the visualizations. In this case, the user defines as execution times “real-time”, “determined” and “overall”. As refresh time, the user defines “5 sec” or “on demand”. Finally, the user specifies the data source that will feed the information for the analysis and selects the categories and measures that will populate the visualizations.

Once the data sources and collections are defined by the users, it is possible to apply our Data Profiling Model. This model will determine, in a semi-automatic manner, the dimensionality, cardinality and type of the data.

We focus on the “sensor values by piece” visualization from the goal-based model (Figure 8). This visualization will require information about the category “sensor” and the measures “value” and “average Value”. Fist, the data profiling tool classifies the independent variable “sensor” as nominal and the dependent variables “value” and “average value” as interval. The dimensionality is set to n-dimensional, due the fact that the user has selected only three variables to visualize. Finally, the cardinality is defined as high because the data contain a large number of items to represent. Overall, the visualization specification obtained through the User Requirements Model and the Data Profiling Model is as follows:

- **Visualization goal**: Comparison
- **Interaction**: Overview
- **User**: Lay
- **Dimensionality**: n-dimensional
- **Cardinality**: High
- **Independent Type**: Nominal
- **Dependent Type**: Interval

Following [33], we are able to automatically translate this visualization specification into the most suitable visualization type. As specified in Section 3.1.3, this process is covered in [11]. In this case, the visualization type that best fits this specification is “multiple line chart”. This whole process is repeated with the rest of the visualizations that compose the model (Figure 8) in order to derive the most suitable visualization type for each specification.

### 4.2. Phase 2—Monitoring of Production Process

Once users have defined the goals of the process and the system has derived the best visualization types (phase 1), it is possible to start monitoring the production process. First, as Figure 5 shows, the Artificial Intelligence model (previously trained) is launched. When the model detects a failure, the sensor analysis process is executed in order to detect whether the fault has been caused by an alteration of the whole machine, or otherwise if the failure has been caused by a specific sensor or group of sensors.

**Figure 9:**
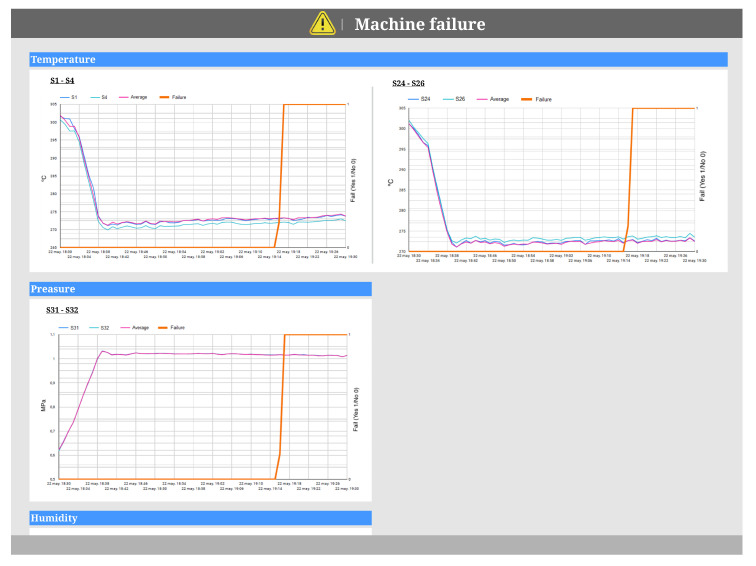
Dashboard of N GSen ALTERED.

In the event that the sensor analysis process detects that the fault has been caused by an alteration of the whole machine (N GSen ALTERED), a dashboard like the one shown in Figure 9 is generated. Following the recommendation of the model shown in Figure 8, a multiple-line chart visualization has been generated to achieve the goal of analyzing damaged parts.

This dashboard represents the overall status of the machine and warns users about the fact that the machine is failing. Thus, all machine sensors are represented, split by the unit of measurement and the localization in the machine. Each visualization represents the evolution of sensor values during the time of the process execution as well as the historical average value of these sensors, which serves as reference to the users. In each visualization, we can see the names of the sensors. The X-axis represents the the date and time when the data were read from the sensors and the Y-axis represents the values of the readings. Additionally, the right side of Y-axis represents whether the process is failing or not.

As we can see in Figure 9, there is no sensor that is out of range, although the machine is failing; therefore, it is possible that the production is not optimal. In order to make decisions and decide whether it is sustainable to stop the production or not, additional visualizations will be necessary.

**Figure 10:**
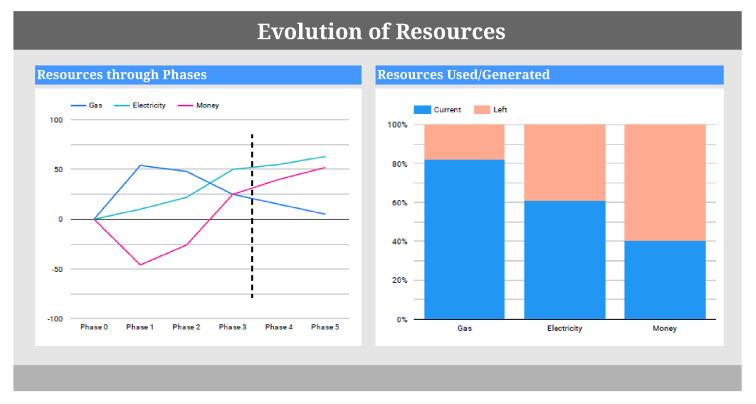
Dashboard showing the evolution of resources.

Figure 10 represents the additional visualizations needed to check the sustainability of the process. On the left side, the visualization represents the use/generation of resources through the phases and marks the stage that the process has reached. This visualization achieves the information goal of the model (Figure 8): “analyze the production through phases”. In this case, the process has almost reached phase 4, and the spent resources are already amortized. Therefore, stopping the production at this moment will only lead to a reduction of the profit; however, if the process were in phase 1, the process would have just begun, and therefore many resources would have been spent and the production would be very low.

On the other hand, the right-side visualization represents the resources that have been used/generated at a specific moment in order to achieve the information goal “analyze the used/generated resources at a certain moment”. This visualization enables users to be more precise in their decisions.

**Figure 11:**
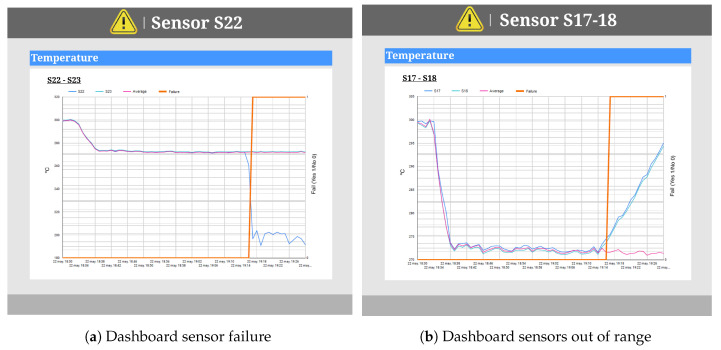
1 Sen/1 GSen FAIL Dashboards.

In the case that the sensor analysis process detects that there is a sensor or group of sensors that is out of range (1 Sen/1 GSen FAIL), one of the dashboards shown in Figure 11 is generated. These dashboards represent the sensors detected as out of range and, for reference, the historical average value of these sensors. Furthermore, the values of sensors located physically close and which do not present anomalies can be included.

Figure 11a,b shows two possible cases that users may face. On the one hand, if the visualization looks like Figure 11a, this means that there is a defective sensor and that it has made an incorrect reading (in this case, sensor S22 is defective). Therefore, the machinery is not affected, and it will not be necessary to stop the production process. The production can continue, and information about the damaged sensor is sent to the AI model to ignore the values of this sensor.

On the other hand, if the system generates a visualization like Figure 11b, this means that there is a problem in this area. It is possible to see in this figure that sensors S17 and S18 show values that are out of the average range. In this case, users should decide if it is a critical moment or if the sensor values are in a critical range; otherwise, if these sensors are not critical to the production, production can continue regardless of this damaged piece.

In the case that it is not a critical moment or if the sensor values are not in a critical range, the production process can continue. However, in this case, we cannot ignore the values of these sensors; a visualization is created in order to allow users to monitor the damaged area. This visualization will enable users to take measures if, at any time, the sensors reach a critical point that may affect the normal operations of the machine or at which the machine would become potentially unsafe for operators, allowing users to stop the machine and proceed to perform maintenance work.

Otherwise, in the case that the values of the sensors are classified as critical to the production process, a dashboard like the one shown in Figure 10 will be presented to users in order for them to decide if it is sustainable or not to stop the production process.

## 5. Limitations

In this section, we summarize the limitations of our work.

- Our proposal has been applied to a specific case study of gas turbines for electricity generation. In principle, the proposal is context-independent, but it should be tested in other production contexts to verify that the results are accurate.
- Our methodology has been developed for non-expert users; however, the user’s domain expertise can be a crucial factor in the definition of more complex dashboards.
- In order to allow users to follow the methodology by themselves, the creation of a CASE tool is necessary.
- Further evaluation of our proposal is required; to this end, we are conducting an empirical evaluation, analyzing the obtained results through the application of our methodology in other production contexts.

## 6. Conclusions and Future Work

Global energy consumption is growing daily, and new lifestyle trends are increasing the need for electricity generation. Industry is benefiting from the rise of technologies such as IoT that enable us to better understand and monitor how production processes are performing. Effective use of these technologies will enable users to take actions aimed at improving both the throughput as well as the sustainability of the process. However, this requires data to exploited from real-time IoT sensors, which is a challenging task due to the size, speed and variety of the data. This is especially cumbersome in industrial IoT devices featuring hundreds of sensors producing measurements which are prone to fail due to several conditions (degradation of sensors, inconsistency among replicated sensors, incomplete data, etc.).

In order to tackle this issue, we have proposed a new methodological approach to monitor industrial machinery through an IoT-based visualization technique. Our approach collects users’ goals and the requirements of the production process, analyzes the incoming data from IoT sensors and automatically derives the most suitable visualization type for each context. It presents a set of visualizations that are intended for non-expert users in data visualization and created by taking into account the level of knowledge of the users. In this sense, our approach makes it easier to visually locate and understand the failures that could arise in a production process and enables users to make the most sustainable decision in each situation.

When this kind of industrial system features AI prediction engines, its complexity is even greater. This is because a neural-network-based AI will commonly not work as a block box and usually provides binary classification results such as “the system is working correctly” or “there will be a problem”. Because of this, it is cumbersome to relate the output of this model with the status of the system’s sensors measurements. However, our approach takes this issue into account by offering visualizations that help users to co-relate AI outputs and sensor’s data, thus enabling them to identify where and when the problem was caused. Otherwise, it is difficult to identify the problematic part within a systems consisting of hundreds of sensors.

Moreover, in order to assess the suitability of our proposal, we have presented a case study based on gas turbines for electricity generation. Our proposal will contribute to the avoidance of unexpected maintenance stops, thus improving the sustainability of the energy-production industry.

As part of our future work, we are working on a further evaluation of our proposal; we are conducting an empirical evaluation, analyzing the results obtained through the application of our methodology. Furthermore, we are working on the creation of a CASE tool in order to facilitate the use of our process, which will be evaluated as in our previous experiments [42].
